# Multicriteria Optimization of the Mechanical Properties in Laminated Seams

**DOI:** 10.3390/ma14112989

**Published:** 2021-05-31

**Authors:** Halina Szafranska, Ryszard Korycki

**Affiliations:** 1 Department of Physicochemistry and Materials Technology, Faculty of Chemical Engineering and Commodity Science, Kazimierz Pulaski University of Technology and Humanities in Radom, Chrobrego Str., 27, 26-600 Radom, Poland; h.szafranska@uthrad.pl; 2 Department of Technical Mechanics, Informatics and Chemistry of Polymer Materials, Faculty of Material Technologies and Textile Design, Lodz University of Technology Zeromskiego, 116, 90-924 Lodz, Poland

**Keywords:** multicriteria optimization, mechanical properties, laminated seams, working clothing

## Abstract

In order to ensure a comprehensive evaluation of laminated seams in working clothing, a series of research was carried out to determine the correlation between the parameters of the seam lamination process (i.e., the temperature, the time, the pressure) and the mechanical properties of laminated seams. The mechanical properties were defined by means of the maximum breaking force, the relative elongation at break and the total bending rigidity. The mechanical indexes were accepted as the measure of durability and stability of laminated seams. The correlation between the lamination process parameters and the final properties of the tested seams in working clothing was proposed using a three-factor plan 3^3^. Finally, the single-criteria optimization was introduced and the objective functional is the generalized utility function U. Instead of three independent optimization problems, the single problem was applied, and the global objective function was a weighted average of partial criteria with the assumed weight values. The problem of multicriteria weighted optimization was solved using the determined weights and the ranges of acceptable/unacceptable values.

## 1. Introduction

Adhesive bonding offers many advantages in good performance and aesthetic appearance as well as speed and economy of the process compared to traditional thread seams. Sealed seams have the barrier properties in respect to heat, moisture and other environmental parameters [1,2,3]. The technological parameters of tapes are usually defined by manufacturers [4,5]. The main problem is to create the seams of the appropriate strength properties using the correlated bonding temperature, pressure, and duration time [6]. The optimization of the selected parameters is difficult in respect of the structure and surface properties of fabric, additional finishing, and characteristics of the tape [6,7].

The influence of ultrasonic welding parameters on bond strength, seam thickness, seam stiffness, and water permeability was discussed in [8]. Different multilayer fabrics and two types of seams (lapped and superimposed) applied for ultrasonic welding were compared with the traditional seam. The quality of seams strongly depends on the quality of surface, tape, and flat joint surface [9].

The fusing/welding methods and their parameters are described in [10] in respect to the thermoplastic adhesives, textile substrates for fusible interlinings, and welding tapes. The characteristics of design and performance of the welded or bonded seams are sensitive to the seam and surface parameters [11] as well as the location of tape on the seam structure [12]. The effects of fiber type, fabric area density, and roller type on ultrasonic tensile properties of nonwoven fabrics are determined in [13]. The ultrasonic seam strength and elongation at break properties of thermal bonded nonwoven fabrics were evaluated to determine the tensile properties.

The works concerning the multicriteria optimization in textile products, structures, or technological processes are relatively rare. Single base injected slub yarn structural parameters (the length, thickness, and frequency) were varied during the preparation of yarn samples [14]. The empirical models relating to slub parameters and fabric abrasion behavior were developed through a backward elimination regression approach.

The parametric approach to complex quality evaluation based on multicriteria decision-making approach for ladies dress materials was discussed in [15]. A complex quality index was estimated with weighted combination of all the contributing parameters.

The multicriteria decision analysis showed good results in the fabrics selection for assuring the sensorial comfort, by using the rating scale delivered by customers [16].

The presented article contains some approaches from previous works of the authors. The laminated seams were analyzed in [17,18] in respect of the selected mechanical properties and applied transfer films. The optimization procedure is usually solved using the optimization functional in general form, the stationarity of Lagrange functional and first-order sensitivity approach [19]. The set of structural parameters (design variables) can be optimized applying the objective functional of unequivocal physical interpretation in case of optimal thickness [20,21] or insulating properties of seams [22]. The same approach can be applied to solve coupled thermo-mechanical problems [23] or identify the structural shape or material properties [24,25]. In case of polymer materials, the elongation appears in the equation defining brittleness B [26,27].

The main goal of this article is to introduce the multicriteria optimization of seam quality in working clothing in respect to the lamination process parameters on a welding press (temperature, time, pressure). The following criteria were selected from the indexes describing the mechanical properties of the seams: (i) the maximum breaking force F [N] for the transverse seam; (ii) the relative elongation at break ε [%]; and (iii) the total bending rigidity G [N m]. Finally, the single-criterion optimization is introduced and the objective function is the generalized utility function U. The function U is a weighted average of partial criteria with the assumed weight values.

Analysis of the available literature has shown that the multicriteria optimization of laminated seams with transfer foil is generally unknown. Thus, the following novelty elements can be identified. (i) Sensitivities of the breaking force, relative elongation, and total bending rigidity were analyzed for the laminated seams in respect to the technological parameters of lamination process. The assumed ranges of parameters were determined on the basis of preliminary tests. (ii) The generalized utility function was introduced as a weighted average of partial criteria functions. Therefore, instead of three independent optimization problems, the single problem and the global objective function were applied. (iii) The multicriteria weighted optimization was solved using the assumed weights and the ranges of acceptable/unacceptable values. (iv) The approach is universal and requires only statistical calculations. Adoption of other satisfactory ranges and/or weights of individual features does not require the new tests of seams.

## 2. Experimental Procedure

To evaluate comprehensively the laminated seams in working clothing, a series of research was carried out to determine the correlation between the lamination process parameters (i.e., the temperature, the time, the pressure) and the mechanical properties of laminated seams.

**Figure 1:**
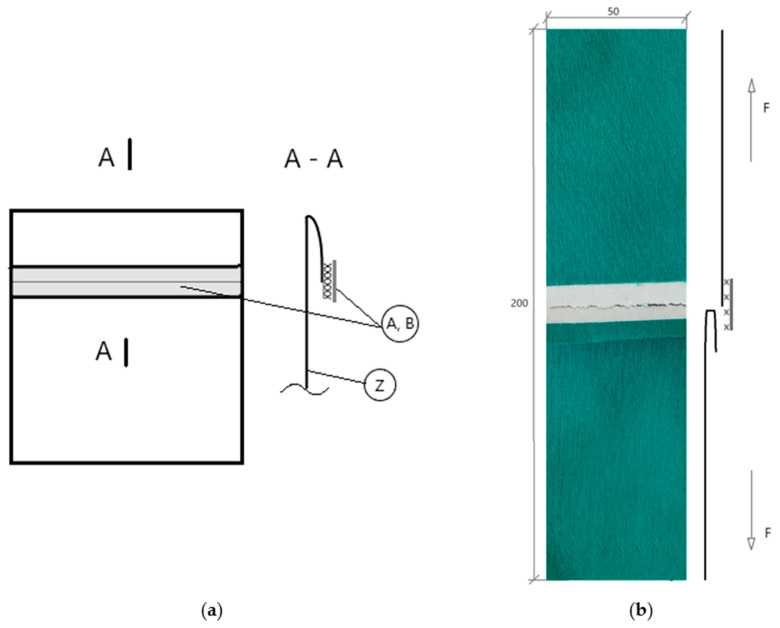
(**a**) Technological project of seam structure in finishing the bottom edges of pocket—the hem laminated seam of 6.03.k type (the cross-section and views); A—StripFlock tape; B—reflective tape; Z—fabric; (**b**) Determination of the strength of seams oriented perpendicular to the direction of force (delamination test).

The research was carried out on a modified version of a hem laminated seam (6.03.k) consisting of cotton fabric Z and two types of tapes placed on the outer side of the product (Figure 1): Tape A, which is of the StripFlock type, where the outer side of the tape is a soft, convex material of velvety texture and the reverse side of the tape is provided with a polyurethane adhesive, and Tape B, which is a reflective thermal transfer film built of light reflecting glass lenses thermally combined with a thermally activated base by means of a polyester adhesive. In comparison to tape A, tape B is characterized by more than double strength and 32-times higher elongation index value.

The seam was designed in such a way that the two fabric layers were shifted with respect to each other, and the tape middle line coincided with the line where two layers of the material joined. Such a seam structure ensures both seam bonding and protecting the edges of the material as well as decoration of the product (Figure 1).

The indexes were analyzed for seams situated in two directions in relation to the acting force, namely: the parallel direction—the so-called longitudinal seams; and the perpendicular direction—the so-called transverse seams.

**Figure 2:**
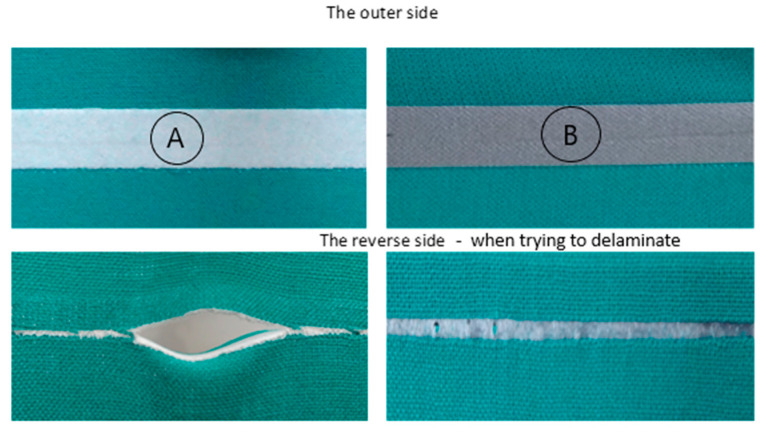
Laminated seams during to delamination tests with tapes (**A**) and (**B**).

Preliminary examinations indicated that during the delamination test no delamination between the fabric and the tape occurred. The adhesive connection between the fabric and the tape was so strong that further acting of the force caused the tape to break. In this way the seam structure was damaged (Figure 2).

The experiment was implemented according to the research program developed on the basis of a three-factorial plan (3^3^) with the following input factors: X_1_—temperature T [°C], X_2_—time t [s], X_3_—pressure *p*∙10^4^ [N/m^2^]. The use of standard experiment plans was preceded by encoding the factors according to the rule presented below.

(1) $$\mathrm{Encoded}\;\mathrm{value}=\frac{\mathrm{mechanical}\;\mathrm{index}-\mathrm{central}\;\mathrm{mechanical}\;\mathrm{index}}{\mathrm{unit}\;\mathrm{of}\;\mathrm{variation}}$$

On the basis of preliminary tests there are determined ranges of factor variability. The encoded values X_1_, X_2,_ X_3_ change within the range <−1,1>. And thus, the lamination temperature of (150, 170, and 190) [°C] corresponded to the values of X_1_ equal to (−1, 0, 1), respectively. Similarly, the values of X_2_ equal to (−1, 0, 1) meant the time s of (10, 15 and 20) [s], respectively. On the other hand the values of X_3_ equal to (−1, 0, 1) meant the pressure of (20, 35 and 50)∙10^4^ [N/m^2^] for seam A and (15, 35 and 55)∙10^4^ [N/m^2^] for seam B, respectively. The central values of the assumed indexes are as follows: temperature T equal to 170 [°C], time t equal to 15 [s] and pressure *p* equal to 35∙10^4^ [N/m^2^].

The following criteria were selected from the indexes describing the mechanical properties of the tested seams: (i) maximum breaking force F [N] for the transverse seam, that is situated in a perpendicular direction to the line of the acting force, (ii) relative elongation at break ε [%]; (iii) total bending rigidity G [N m] [17,18].

## 3. Statistical Analysis

The criteria were selected in respect of the seam location in the clothing and its functional aspect. Estimation of the regression function describing the relationships between the considered factors and examined properties was carried out with the use of the SAS computer program. The results obtained are used for the regression analysis, which allowed to estimate, with the use of polynomials of degree 2, the correlations between the examined factors and the property in question. The value of the regression coefficients was estimated according to the Student’s test and adequacy of the developed model according to the value of the multiple correlation coefficient R^2^.

The models described the lamination process adequately, which is proved by the values of the correlation coefficient R^2^ being within the range from 0.56 to 0.78 [17,18].

Owing to the establishment of correlations it is possible to predict changes in the values of the maximum breaking force index, relative elongation index and total bending rigidity index for the laminated seams A and B subject to the product and given seam function requirements. However, the changes in value can be predicted for a single index.

The aim of this article is to evaluate comprehensively the selected seams equipped with tapes A and B using the multicriteria statistical optimization and the generalized utility function *U*, while all three analyzed indexes (i.e., the criteria F, ε, G) change simultaneously. The generalized utility function U is defined as the weighted exponential mean of the partial utilities *U_i_*.

(2) $$U^{}=\exp\left[-\exp\sum_{i=1}^{m}w_{i}\left(-\frac{y^{\left(i\right)}-y_{G}^{\left(i\right)}}{y_{L}^{\left(i\right)}-y_{G}^{\left(i\right)}}\right)\right],\;\sum_{i=1}^{m}w_{i}=1,$$

where *w_i_* are the weights attributed to the particular criteria *y*^(*i*)^; 0 ≤ *w_i_* ≤ 1; *i* = 1, 2, *m* = 3.

**Figure 3:**
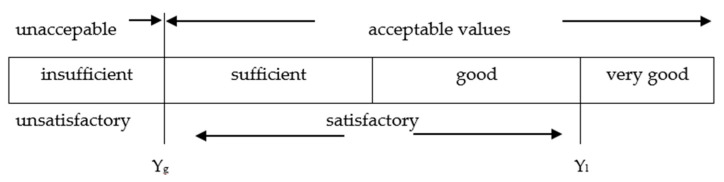
Selection of the range of satisfactory values for the criterion Y.

The values of generalized utility *U* are within the range from 0 to 1; the greater the value, the more favorable properties of the tested seam. The particular value of the generalized utility allows to evaluate the tested seam in regard to the entire set of adopted criteria with their importance. The values of all considered criteria were expressed therefore on a dimensionless scale. The scale was determined by means of a range of satisfactory values for each criterion, limited by the worst values *y_g_*^(*i*)^ and the best values *y_l_*^(*i*)^, Figure 3.

A range of values can be selected as satisfactory in case of the effective application of the seam in working clothing, for example finishing the bottom edges of skirts, aprons, trousers, sleeves, or the upper edge of pockets. Regardless of the ranges of satisfactory values, the particular criteria are determined by the non-negative weighting factors, reflecting the importance of each criterion. The sum of the weighting factors should always be equal to 1.

Based on the technical requirements [28] and consultations with production engineers in clothing factories, the ranges of satisfactory values and weights of particular criteria were adopted, as shown in Table 1. The optimal seams are created for the maximum values of all criteria.

According to the analyzed method of multi-criteria statistical optimization, the most important index is the maximum breaking force F [N] with the greatest weight equal to 0.5. The other indexes G [N m] and ε [%] are characterized by lower weights equal to 0.3 and 0.2. The sum of attributed weights is equal to 1, as shown in Table 1.

Assuming the appropriate ranges of satisfactory values and the weights of particular criteria, the utility function U is determined according to the experiment.

Table 2 contains the regression equations formulated on the basis of the accepted ranges of satisfactory values, which allows to determine the sensitivity of the mechanical properties of the tested seams to the variable factors. The obtained models described the lamination process adequately, which is proved by the value of the correlation coefficient R^2^.

Table 3 includes the values of utility function for A and B seams, tested at varying values of temperature, time, and pressure. The generalized utility U is described by values being within the range from 0 to 1. Numbers close to 0 correspond to the particularly unfavorable values of the feature *y*^(*i*)^, whereas numbers close to 1 correspond to the most favorable values of the feature.

**Figure 4:**
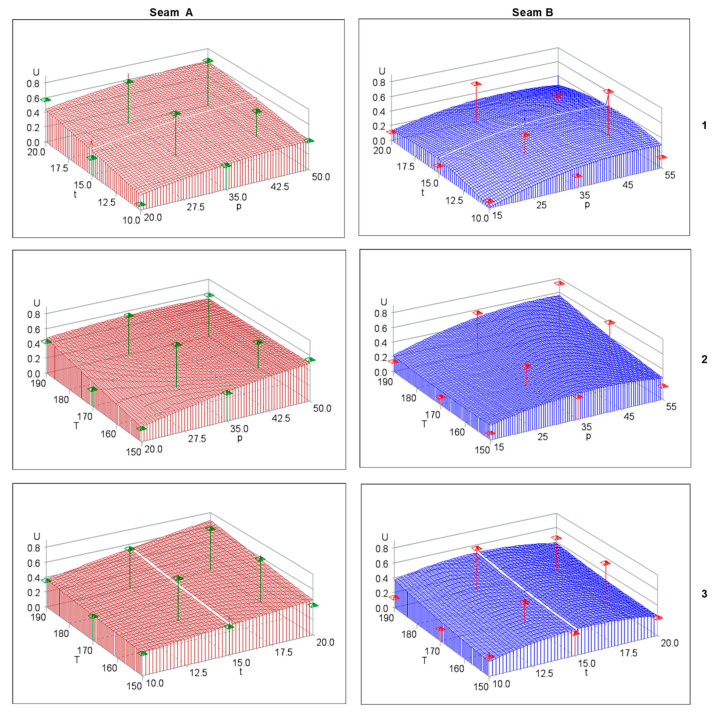
Response surface—diagram of the regression function: utility function vs. lamination process parameters: **1**—time t and pressure *p* at constant temperature T = 170 [°C]; **2**—temperature T and pressure *p* at constant time t = 15 [s]; **3**—temperature T and time t at constant pressure *p* = 35 × 10^4^ [N/m^2^].

Substituting the numbers into Equation (1), we obtain a set of values *y*^(*i*)^ defined as satisfactory in the range from 0.37 to 0.69. In this range, the utility function is the most sensitive to change in the value of an individual feature. The diagram of the regression function: utility function versus the lamination process parameters (temperature T, time t, pressure *p*) was determined for the seams A and B, as shown un Figure 4.

All values of statistical significance *p* less than 0.05 are statistically significant and confirm the essential influence of a given factor on the individual feature.

The response surfaces of the generalized utility function U in case of seam A are very similar, irrespective of the analyzed factor (the left column of Figure 4). Thus, the utility function is also statistically sensitive to all individual factors: temperature T, time t and pressure *p*. This is adequately proved by the low values of statistical significance according to Table 4. The seam A is particularly sensitive to the time factor t, which determines the values of statistical significance *p* equal to 0.0004 (Table 4).

The response surfaces of the generalized utility function U are quite different in the case of seam B and any individual factor (the right column of Figure 4). Statistically speaking, the factors of temperature T and time t are statistically insignificant. This is confirmed again by the values according to Table 4. The factor of pressure *p* is statistically significant and equal to 0.0018 for seam A, and 0.0286 for seam B. The analysis of the response surface (Figure 4) allows to predict that the highest value of utility function for the seam B is achieved for the maximum values of temperature and pressure.

The values of utility function U determined on the basis of regression equations for the seams A and B are within the following ranges (Table 3): (i) the seam A from 0.09 to 0.66; (ii) the seam B from 0.10 to 0.74.

The wide range of the generalized utility function contains both acceptable and unacceptable values, which excludes the application of any chosen variant of tested variables. The clothing manufacturers and users are interested in the maximum possible durability/stability of the seam in clothing. Therefore, the variants of variable factors were selected, which ensures that the function U is at least in the range of satisfactory values <0.37 ÷ 0.69>.

The variants for seam A are the following:

T (150, 170, 190) [°C];

t (15, 20) [s];

*p* (20, 35, 50) × 10^4^ [N/m^2^].

In the case of seam B, the values are listed below:

T = 190 [°C], t = 15 [s], *p* = 55 × 10^4^ [N/m^2^];

T = 190 [°C], t = 10 [s], *p* = 55 × 10^4^ [N/m^2^];

T = 170 [°C], t = 15 [s], *p* = 55 × 10^4^ [N/m^2^];

T = 190 [°C], t = 15 [s], *p* = 35 × 10^4^ [N/m^2^].

In practice, in the case of seam A it is possible to apply more variants of variable factors than for seam B.

The maximum values of the utility function U were obtained under the following conditions of the lamination process for seam A.

U = 0.66 for T = 190 [°C], t = 20 [s], *p* = 50 × 10^4^ [N/m^2^] and *p* = 20 × 10^4^ [N/m^2^];

U = 0.64 for T = 170 [°C], t = 20 [s], *p* = 50 × 10^4^ [N/m^2^].

The maximum values of the function U for the seam B are obtained in the conditions.

U = 0.74 for T = 190 [°C], t = 15 [s], *p* = 55 × 10^4^ [N/m^2^];

U = 0.62 for T = 170 [°C], t = 15 [s], *p* = 55 × 10^4^ [N/m^2^];

U = 0.61 for T = 190 [°C], t = 10 [s], *p* = 55 × 10^4^ [N/m^2^].

The generalized utility function allowed to evaluate the examined seams A and B in regard to all selected features, taking into account their importance. The regression analysis showed the significant influence of the factors: temperature T, time t, pressure *p* during the lamination process on the value of the utility function U in seam A. On the other hand, the influence of temperature T, time t on the value of the utility function in seam B was statistically insignificant. This most likely causes the number of variants of variable factors to ne smaller in the range of satisfactory values U <0.37 ÷ 0.69>. However, the combination of factor values led to the high/very good utility of U equal to 0.74.

## 4. Conclusions

Examinations of laminated seams in cotton working clothing allowed to determine the range of variable parameters (that is, temperature T [°C], time t [s], pressure *p* [N/m^2^]) of the lamination process on a welding press, which enables to expect the most favorable values of mechanical indexes important for the clothing manufacturer.

The impact of lamination process parameters (temperature T, time t, pressure *p*) on the durability and stability of laminated seams A and B was assessed comprehensively. The obtained mathematical models of the influence of tapes A and B on the generalized utility function U allow to predict changes in the mechanical properties of seams caused by temperature, time, and pressure.

Based on the analysis of the obtained results of generalized utility function U, the selection of individual test variants was justified, which ensure the best durability/stability of laminated seams in cotton working clothing.

The great advantage of the method adopted is that, starting from the three-criteria optimization in respect of the maximum breaking force F, the relative elongation at break ε and the total bending rigidity G, we formulate at the last stage the single-criterion optimization. The generalized utility function integrates all requirements into the single indicator U.

The multicriteria weighted optimization was solved using the assumed weights and the ranges of acceptable/unacceptable values. Adoption of other satisfactory ranges and/or weights of individual features does not require the new examinations of seams, but only a repetition of statistic calculations.

The working conditions of clothing determine the selection of global optimization criterion. The optimization criterion of the maximum mechanical properties was introduced in the article: the higher the values of all partial criteria, the more optimal the seam structure in respect to the mechanical properties. Different working conditions of clothing require a change of the applied criterion. Sometimes a softness of seams is recommended in clothing products. This means that the accepted ranges of satisfactory values according to Table 1 for the bending stiffness G should be changed in the descending direction (e.g., from 120 to 55). Applying the results of one research cycle, many potential applications can be considered, and the solution can be always determined in regard to the selected optimization criterion.

Summarizing, the method of multicriteria statistical optimization is a promising tool for predicting changes in the performance properties of laminated seams.

## Figures and Tables

**Table 1:** Ranges of satisfactory values and weights attributed to the particular criteria for tapes A and B.

Index	Range of Satisfactory Values		Weight
	The Worst Value	The Best Value	
F [N]	160	200	0.5
G [N m]	55	120	0.3
ε [%]	6.5	9.8	0.2

**Table 2:** Regression equations describing utility function U vs. lamination process parameters (T, t, *p*) for seam A and seam B.

Seam Type	Regression Equations of Utility Function U	R^2^
A	U = − 0.798132 − 0.000064429X_1_ − 0.037870X_2_ + 0.053329X_3_ + 0.000014970X_1_^2^ + 0.0004000X_1_X_2_ − 0.000213X_2_^2^ − 0.000207X_1_X_3_ + 0.000008739X_2_X_3_ − 0.000174X_3_^2^	0.82
B	U = 0.005673 − 0.017101X_1_ + 0.180285X_2_ + 0.002888X_3_ + 0.000051691X_1_^2^ − 0.000148X_1_X_2_ − 0.004584X_2_^2^ + 0.000157X_1_X_3_ − 0.000389X_2_X_3_ − 0.000260X_3_^2^	0.56

**Table 3:** Influence of lamination process parameters on value of utility function for seams A and B.

Nr	X1	X2	X3	Temp.	Time	Pressure		Utility U	
				T [°C]	t [s]	*p*∙10^4^ [N/m^2^]		Tape A	Tape B
						Tape A	Tape B		
1	−1	−1	−1	150	10	20	15	0.214	0.096
2	−1	−1	0	150	15	20	15	0.186	0.103
3	−1	−1	1	150	20	20	15	0.179	0.109
4	0	−1	−1	170	10	20	15	0.090	0.109
5	0	−1	0	170	15	20	15	0.242	0.117
6	0	−1	1	170	20	20	15	0.585	0.126
7	1	−1	−1	190	10	20	15	0.335	0.116
8	1	−1	0	190	15	20	15	0.437	0.151
9	1	−1	1	190	20	20	15	0.655	0.157
10	−1	0	−1	150	10	35	35	0.298	0.268
11	−1	0	0	150	15	35	35	0.384	0.309
12	−1	0	1	150	20	35	35	0.409	0.251
13	0	0	−1	170	10	35	35	0.332	0.174
14	0	0	0	170	15	35	35	0.582	0.268
15	0	0	1	170	20	35	35	0.595	0.544
16	1	0	−1	190	10	35	35	0.366	0.149
17	1	0	0	190	15	35	35	0.549	0.569
18	1	0	1	190	20	35	35	0.588	0.473
19	−1	1	−1	150	10	50	55	0.353	0.179
20	−1	1	0	150	15	50	55	0.562	0.193
21	−1	1	1	150	20	50	55	0.618	0.262
22	0	1	−1	170	10	50	55	0.400	0.160
23	0	1	0	170	15	50	55	0.363	0.616
24	0	1	1	170	20	50	55	0.644	0.143
25	1	1	−1	190	10	50	55	0.383	0.610
26	1	1	0	190	15	50	55	0.595	0.743
27	1	1	1	190	20	50	55	0.660	0.147

**Table 4:** Comparison of values of statistical significance *p* for the factors T, t, *p* analyzed.

Factor	Generalized Utility Function U	
	Seam A	Seam B
Temperature T [°C]	0.0047	0.2309
Time t [s]	0.0004	0.3929
Pressure *p*∙10^4^ [N/m^2^]	0.0018	0.0286

## Data Availability

Not applicable.
